# Multiparametric MRI texture analysis in prediction of glioma biomarker status: added value of MR diffusion

**DOI:** 10.1093/noajnl/vdab051

**Published:** 2021-04-08

**Authors:** Shingo Kihira, Nadejda M Tsankova, Adam Bauer, Yu Sakai, Keon Mahmoudi, Nicole Zubizarreta, Jane Houldsworth, Fahad Khan, Noriko Salamon, Adilia Hormigo, Kambiz Nael

**Affiliations:** 1 Department of Diagnostic, Molecular and Interventional Radiology, Icahn School of Medicine at Mount Sinai, New York, New York, USA; 2 Department of Pathology, Icahn School of Medicine at Mount Sinai, New York, New York, USA; 3 Department of Radiology, Kaiser Permanente Fontana Medical Center, Fontana, California, USA; 4 Institute for Health Care Delivery Sciences, Icahn School of Medicine at Mount Sinai, New York, New York, USA; 5 Department of Radiological Sciences, David Geffen School of Medicine at University of California Los Angeles, Los Angeles, California, USA

**Keywords:** glioma, MR diffusion, multiparametric MRI, radiogenomics, texture analysis

## Abstract

**Background:**

Early identification of glioma molecular phenotypes can lead to understanding of patient prognosis and treatment guidance. We aimed to develop a multiparametric MRI texture analysis model using a combination of conventional and diffusion MRI to predict a wide range of biomarkers in patients with glioma.

**Methods:**

In this retrospective study, patients were included if they (1) had diagnosis of gliomas with known *IDH1*, *EGFR*, *MGMT*, *ATRX*, *TP53*, and *PTEN* status from surgical pathology and (2) had preoperative MRI including FLAIR, T1c+ and diffusion for radiomic texture analysis. Statistical analysis included logistic regression and receiver-operating characteristic (ROC) curve analysis to determine the optimal model for predicting glioma biomarkers. A comparative analysis between ROCs (conventional only vs conventional + diffusion) was performed.

**Results:**

From a total of 111 patients included, 91 (82%) were categorized to training and 20 (18%) to test datasets. Constructed cross-validated model using a combination of texture features from conventional and diffusion MRI resulted in overall AUC/accuracy of 1/79% for *IDH1*, 0.99/80% for *ATRX*, 0.79/67% for *MGMT*, and 0.77/66% for *EGFR*. The addition of diffusion data to conventional MRI features significantly (*P* < .05) increased predictive performance for *IDH1*, *MGMT*, and *ATRX*. The overall accuracy of the final model in predicting biomarkers in the test group was 80% (*IDH1*), 70% (*ATRX*), 70% (*MGMT*), and 75% (*EGFR*).

**Conclusion:**

Addition of MR diffusion to conventional MRI features provides added diagnostic value in preoperative determination of IDH1, MGMT, and ATRX in patients with glioma.

**Key Points**

- Combination of T1c+, FLAIR and diffusion texture features can predict several glioma biomarkers.
- Addition of diffusion MRI features significantly improved prediction for *IDH1*, *MGMT* and *ATRX*.
- No texture features were identified to predict *PTEN* and *TP53*.

**Importance of the Study**

In patients with glioma, our knowledge about the status of tumoral biomarkers has changed our approach in terms of histopathological classification with provided prognostic and therapeutic implications. Prior studies have assessed glioma biomarkers statuses using texture data from either conventional or diffusion MR imaging with some success. This study aims to construct a multiparametric model combining radiomic data from T1c+, FLAIR, and diffusion MR features to predict individual glioma biomarker statuses. Addition of MR diffusion to conventional MRI features provides added diagnostic value in preoperative determination of IDH1, MGMT, and ATRX in patients with glioma. Early and noninvasive recognition of these biomarkers would help neuro-oncologists to construct a more specific prognostic and treatment plan for patients with glioma.

Gliomas are the most common primary brain neoplasms accounting for roughly 40%–50% of all malignant primary central nervous system tumors.^1^ Despite advances in diagnosis and treatment, the prognosis of patients with high-grade gliomas or glioblastomas (GBM) remain dismal with 5-year survival rate of 5%–10%.^2^ As molecular phenotypes have been found to successfully predict prognosis and guide treatment options, there is increasing emphasis on identification of these biomarkers to better understand the pathophysiology of gliomas and to explore more specific targeted treatment options.

The earliest biomarkers to make up the genetic hallmarks of GBMs were upregulation of *EGFR*,^3^ mutations in *TP53*,^4^ and mutations in *PTEN*.^5^ Since then, methylation of the *MGMT* gene promoter was found to be a predictor of treatment outcome of temozolomide and radiotherapy.^6^ More recently, *IDH1* mutation was found to be an independent positive prognostic biomarker with significantly longer progression-free survival and better treatment outcome for chemotherapy plus radiation compared to *IDH1* wildtype.^7^ The WHO Classification of 2016 now further categorizes GBMs by IDH status and encourages routine testing for mutational status. Radiogenomic mapping has emerged as a promising noninvasive tool for predicting these biomarkers. To date, using conventional MRI sequences (T1c+/FLAIR), several investigators have found radiomic associations with *IDH1* mutation,^8,9^*MGMT* methylation status,^10,11^*EGFR* amplification,^12,13^*ATRX* mutation,^14,15^*PTEN* deletion,^16^ and *TP53* mutation^17^ with varying success. The ability to predict biomarker status via radiomics noninvasively is invaluable as large tissue specimens are often needed for accurate histopathological diagnosis and there are limited laboratories that can perform these tests. Radiomics may have the potential to provide complimentary information to overcome some of the limitations of histologic assessment as related to insufficient sampling or tumor heterogeneity.^18^ Furthermore, presurgical identification of these biomarkers can facilitate patient counseling and contribute to surgical planning and optimal patient management when complete molecular characterization is not possible.

In this study, we aimed to develop a multiparametric MRI texture analysis model using a combination of conventional (T1c+/FLAIR) and diffusion MRI to predict individual glioma biomarkers. We specifically investigated the potential added value of combining MR diffusion radiomics with conventional MRI in order to improve the accuracy of our predictive model.

## Material and Methods

This retrospective study was approved by an institutional review board and informed consent was waived. Patients with initial diagnosis of glioma between January 2016 to September 2018 were reviewed. A total of 151 patients were reviewed (Supplementary Figure 1). Patients were included if they (1) had diagnosis of gliomas with known *IDH1*, *EGFR*, *MGMT*, *ATRX*, *PTEN*, and *TP53* status from surgical pathology and (2) had preoperative MRI including FLAIR, T1c+, and diffusion within 30 days of biopsy or surgical resection. Patients were excluded if they had insufficient MR image quality (motion artifact, *n* = 8), prior surgeries involving the tumoral bed (*n* = 8) or treated with radiotherapy previously (*n* = 4). In addition, 20 patients were excluded due to lack of preoperative diffusion imaging. This yielded a final cohort of 111 patients. A total of 91 patients (82%) were categorized as training dataset for model development and 20 patients (18%) as testing dataset for assessment of predictive accuracy.

### Histopathological Data

Tissue samples were obtained from patients undergoing targeted tissue biopsy or resection, as part of routine clinical care and diagnostic neuropathology and molecular evaluation. Immunohistochemistry was used to detect mutant status of *IDH1* (specifically IDH1^R132H^ immunoreactivity) and *ATRX* (loss of nuclear staining). Chromogenic in situ hybridization was used to assess *EGFR* amplification signal. Targeted next-generation sequencing was used to detect *PTEN* and *TP53* mutational status. Pyrosequencing of bisulfite-treated genomic DNA (CpG sites 74–78, QIAGEN) was used to detect *MGMT* promoter methylation status.

### Image Acquisition

MR imaging was obtained using 7 MRI scanners (2 Skyra 3T and 2 Aera 1.5T from Siemens Healthineers, Erlangen Germany; 2 Signa 1.5T and one Discovery 3T from GE Healthcare) within our Radiology Department. Image acquisition was performed using a standardized preoperative brain tumor MRI protocol within our radiology department including: FLAIR (TR/TE/TI, 8000–12,000/98–130/2400–2700 ms, voxel size: 0.5 × 0.5 × 1 mm^3^), DWI (TR/TE: 4025–4600/65–82 ms, with *b* values of 0 and 1000 s/mm^2^, voxel size: 0.9 × 0.9 × 5.0 mm^3^) and post-contrast T1W imaging (TR/TE, 600–1800/9–19 ms, voxel size: 0.5 × 0.5 × 1 mm^3^). A total volume of 0.1 mmol/kg of gadobenate dimeglumine was injected intravenously for post-contrast T1W imaging.

### Image Analysis

Using our training dataset (*n* = 91 patients), image analysis was performed by a commercially available FDA-approved software (Olea Sphere software, Olea Medical SAS). Automatic preprocessing was standardized for each case involving intensity normalization, resampling, and discretization. Since MR images were obtained using different MRI scanners from 2 vendors and with different magnetic fields, a normalization step was implemented to normalize images by centering at the mean with standard deviation using all gray values in the image. The resampling grid was aligned to the input origin enabling in-plane resampling. Size and number of bins was set to 25 and 64, respectively, for every case standardizing the process of making histogram and discretion of the image gray level. T1c+, FLAIR, and diffusion images (ADC/b1000) were coregistered on each examination using a 6-df transformation and a mutual information cost function.

Tumor segmentation was performed manually on every slice that the tumor was visualized using FLAIR images. This was performed by a trained radiologist and under supervision of a board certified neuroradiologist. Subsequently, a VOI was generated encompassing the entire region of FLAIR hyperintensity and overlaid onto coregistered T1c+ and diffusion datasets for radiomic texture analysis (Supplementary Figure 2).

A total of 92 radiomic features were assessed. These included 19 first-order metrics, such as the mean, standard deviation, skewness, and kurtosis, and second-order metrics including 23 gray level run length matrix (GLCM), 16 gray level run length matrix (GLRLM), 15 gray level size zone matrix (GLSZM), 5 neighboring gray tone difference matrix (NGTDM), and 14 gray level dependence matrix (GLDM). Details of the definitions and calculations of these features have previously been reported.^19–23^ Texture feature extraction through Olea sphere software was in compliance with the image biomarker standardization initiative with the above 92 features categorized into (1) histogram features, which included grey intensity or brightness information of the lesion, (2) form factor features, which describe the shape and compactness of the lesions, and (3) texture features, which includes the remainder of the second-order metrics as above, and has been cited in prior studies.^24,25^

### Statistical Analysis

Statistical analysis was performed using Matlab R2019b and Statistics and Machine Learning Toolbox (The MathWorks, Inc.) and SAS 9.4M6 (TS1M6) 2020 (SAS Institute Inc.). Ninety-two texture features were obtained from each imaging sequence (T1c+, FLAIR, ADC, and b1000) resulting in a total of 368 features for each patient in our training cohort. One-way analysis of variance for each imaging parameter (*n* = 368) was performed with each biomarker (*IDH1* [wildtype vs mutated], *MGMT* [methylated vs unmethylated], *EGFR* [amplified vs nonamplified], *ATRX* [wildtype vs mutated], *TP53* [wildtype vs mutated], and *PTEN* [wildtype vs mutated] as the independent variable. From the texture feature means that differed with statistical significance (*P* < .05) between biomarker positivity, Least Absolute Shrinkage and Selection Operator (LASSO)^26^ regularization was employed to select contributing variables to the models, thereby reducing potential risk of overfitting and increased interpretation. The significant contributing variables were then entered into a stepdown logistic regression analysis. A stepwise method was used to avoid collinearity because redundant variables were omitted. A 10-fold cross-validation scheme was used for evaluation of the training cohort, where 90% of the data was randomly assigned into the training cohort and 10% used for validation. This process was repeated 10 times.

Receiver-operating characteristic (ROC) curves were generated and area under the curve (AUC) was estimated for cross-validated models utilizing conventional MRI (T1c+, FLAIR) features first and then by addition of MRI-diffusion features. A comparative analysis between ROCs (conventional vs conventional + diffusion) was performed using nonparametric methods described by DeLong et al.^27^ Optimal thresholds were determined to maximize sensitivity and specificity for each biomarker utilizing the Youden’s index. The final constructed model was applied to a testing dataset to calculate the accuracy of biomarker prediction.

## Results

### Clinical Characteristics of Patient Population

Our final patient cohort consisted of a total of 111 patients (Table 1). The mean ± standard deviation of age (years) was 57 ± 15 with median of 59. Sixty-four patients were male and 47 were female. There were a total of 92 patients with GBM and 19 patients with lower-grade glioma including grade II glioma (*n* = 7) and grade III glioma (*n* = 12). There were 19 nonenhancing tumors and 92 enhancing tumors (Table 1). The demographic data including age, sex, tumor grade, and enhancement status are grouped based on biomarker mutation status and summarized in Table 1.

**Table 1.** Demographic Data for Each Glioma Biomarker

	Status	*N* (%)	Age (mean ± SD)	Sex (M/F)	Enhancement (%)	WHO Grade (II/III/IV)
Total		111 (100)	57 ± 15	64/47	92 (83)	7/12/92
*IDH1*						
	Wild-type	88 (79)	61 ± 11*	48/40	86 (98)	0/0/88
	Mutant	23 (21)	40 ± 11	16/7	6 (26)	7/12/4
*MGMT*						
	Nonmethylated	59 (47)	58 ± 12	30/29	53 (90)	4/2/53
	Methylated	52 (53)	55 ± 15	34/18	39 (75)	3/10/39
*EGFR*						
	Wild-type	76 (68)	56 ± 14	39/37	59 (78)	7/12/57
	Amplified	35 (32)	59 ± 12	25/10	33 (94)	0/0/35
*ATRX*						
	Wild-type	92 (83)	60 ± 11*	50/42	86 (93)	3/2/87
	Mutant	19 (17)	39 ± 9	14/5	6 (32)	4/10/5
*TP53*						
	Wild-type	84 (76)	59 ± 13	44/40	74 (88)	4/6/74
	Mutant	27 (24)	47 ± 15	20/7	18 (67)	3/6/18
*PTEN*						
	Wild-type	100 (90)	56 ± 14	60/40	81 (81)	7/12/81
	Mutant	11 (10)	63 ± 10	4/7	11 (100)	0/0/11

*Statistically significant (*P* < .05).

### Model Development in Training Cohort (*n* = 91)

#### IDH1.—

**Figure 1.**
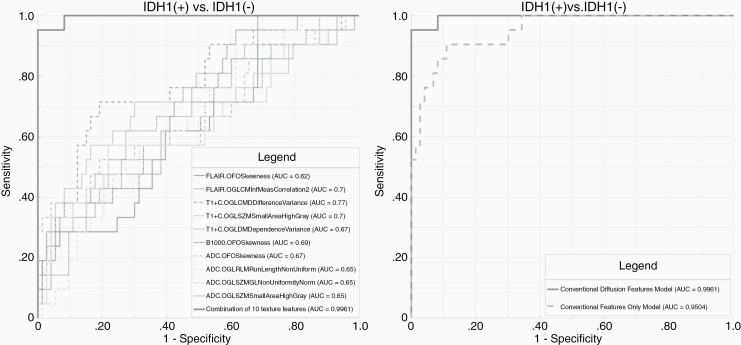
Receiver-operating characteristic (ROC) curves for prediction of *IDH1* mutational status. (A) Combined model constructed with a combination of 5 conventional and 5 diffusion MR texture features with AUC: 1.0. (B) Comparative analysis of ROCs shows significant (*P* = .03) improvement in prediction of *IDH1* status in the combined model (conventional + diffusion MR) in comparison to the conventional only model. Parameters (*n* = 10) included in the final model are GLCM informal measure correlation 2 (T1c+), first-order skewness (T1c+), GLCM difference variance (T1c+), GLSZM small area high gray (T1c+), GLDM dependence variance (T1c+), first-order skewness (b1000), first-order skewness (ADC), GLRLM run length non-uniformity (ADC), GLSZMGL non-uniformity normalized (ADC), and GLSZM small area high gray (ADC).

Following LASSO regularization and logistic regression analysis, a total of 10 texture features from conventional MR imaging remained as significant contributors in our predictive model with resultant AUC of 0.95 (Supplementary Table 1). After addition of diffusion data, a combination of 5 conventional and 5 diffusion MR features remained as significant contributors (Supplementary Table 2), improving the AUC to 1.0 (Figure 1). The addition of diffusion data significantly (*P* = .03) increased the predictive performance for *IDH1* (Table 2). The AUC, sensitivity, specificity, and threshold for the conventional versus conventional + diffusion model are summarized in Table 2.

**Table 2.** Prediction Performance for Glioma Biomarkers Through Optimal Combination of Conventional and Diffusion MR Features

	T1c+/FLAIR	T1c+/FLAIR + Diffusion	ROC Comparison
	(AUC/Sens/Spec/Acc/threshold)	(AUC/Sens/Spec/Acc/threshold)	*P* value
*IDH1*	0.95/67/85/76/0.33	1/87/71/79/0.76	.03
*MGMT*	0.64/41/61/51/0.56	0.79/70/65/67/0.51	.006
*ATRX*	0.92/78/76/77/0.1	0.99/72/88/80/0.64	.01
*EGFR*	0.77/74/62/68/0.3	0.83/65/68/66/0.38	.17
*TP53*	*	Noncontributory	NA
*PTEN*	*	Noncontributory	NA

*No variables remained as significant contributors following logistic regression analysis.

#### MGMT.—

**Figure 2.**
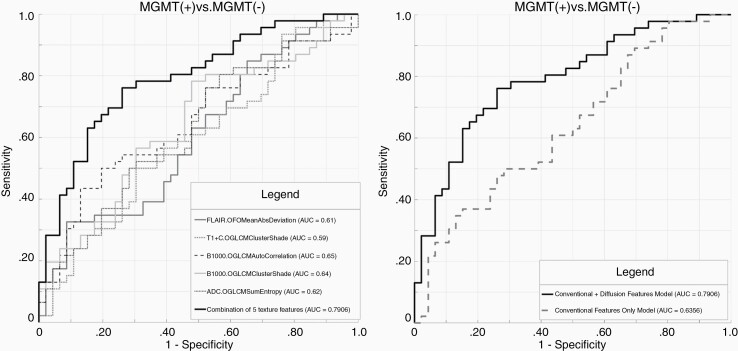
Receiver-operating characteristic (ROC) curves for prediction of *MGMT* methylation status. (A) Combined model constructed with a combination of 2 conventional and 3 diffusion MR texture features with AUC: 0.79. (B) Comparative analysis of ROCs shows significant (*P* = .006) improvement in prediction of *MGMT* methylation status in combined model (conventional + diffusion MR) in comparison to conventional only model. Parameters (*n* = 5) included in the final model are first-order mean absolute deviation (FLAIR), GLCM cluster shade (T1c+), GLCM autocorrelation (b1000), GLCM cluster shade (b1000), and GLCM sum entropy (ADC).

Following LASSO regularization and logistic regression analysis, only a total of 2 conventional imaging features remained as significant contributors with overall AUC of 0.64 (Supplementary Table 1). With the addition of diffusion features, a combination of 2 conventional and 3 diffusion MR features (Supplementary Table 2), resulted in significant (*P* = .006) improvement in predicting model, increasing the AUC to 0.79 (Figure 2). The AUC, sensitivity, specificity, and threshold for the conventional versus conventional + diffusion model are summarized in Table 2.

#### ATRX.—

**Figure 3.**
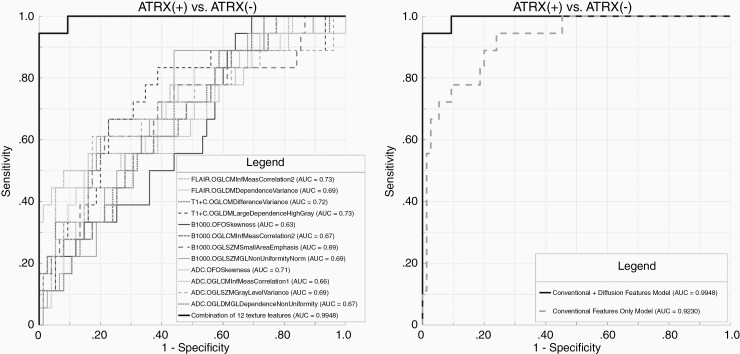
Receiver-operating characteristic (ROC) curves for prediction of *ATRX* mutational status. (A) Combined model constructed with a combination of 4 conventional and 8 diffusion MR texture features with AUC: 0.99. (B) Comparative analysis of ROCs shows significant (*P* = 0.01) improvement in prediction of *ATRX* mutational status in combined model (conventional + diffusion MR) in comparison to conventional only model. Parameters (*n* = 12) included in the final model are GLCM informal measure correlation 2 (FLAIR), GLDM dependence variance (FLAIR), GLCM difference variance (T1c+), GLDM large dependence high gray (T1c+), first-order skewness (b1000), GLCM informal measure correlation 2 (b1000), GLSZM small area emphasis (b1000), GLSZMGL non-uniformity normalized (b1000), first-order skewness (ADC), GLCM informal measure correlation 1 (ADC), GLSZM gray level variance (ADC), and GLDMGL dependence non-uniformity (ADC).

A total of 6 conventional imaging features remained as significant contributors in our predictive model with resultant AUC of 0.92 (Supplementary Table 1). After incorporating diffusion features, the final model consisted of a combination of 4 conventional and 8 diffusion features (Supplementary Table 2). Addition of diffusion features resulted in significant (*P* = 0.01) improvement in predictive performance with an AUC of 0.99 (Figure 3). The AUC, sensitivity, specificity, and threshold for the conventional versus conventional + diffusion model are summarized in Table 2.

#### EGFR.—

**Figure 4.**
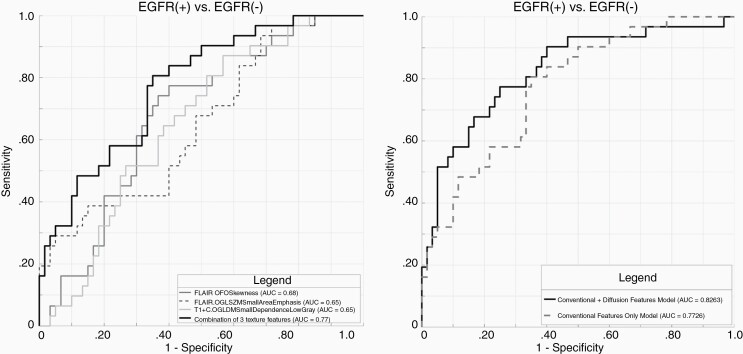
Receiver-operating characteristic (ROC) curves for prediction of *EGFR* amplification. (A) Final model was constructed with 3 conventional imaging features with AUC: 0.77. (B) Addition of diffusion features did not result in significant (*P* = .17) improvement in prediction of *EGFR* amplification. Parameters (*n* = 3) included in the final model are first-order skewness (FLAIR), GLSZM small area emphasis (FLAIR), and GLDM small dependence low gray (T1c+).

A total of 3 conventional imaging features remained as significant contributors in our predictive model with resultant AUC of 0.77 (Supplementary Table 1). After integrating diffusion features, there was no statistically significant (*P* = .17) improvement in diagnostic performance (Figure 4). The AUC, sensitivity, specificity, and threshold for the model constructed from conventional MRI features are summarized in Table 2.

#### TP53.—

Following LASSO regularization and logistic regression analysis, no texture features remained as significant contributors in our predictive model.

#### PTEN.—

Following LASSO regularization and logistic regression analysis, no texture features from conventional MRI remained as significant contributors in our predictive model.

### Independent Testing Cohort (*n* = 20)

The predictive accuracy of the validated models was tested in a total of 20 patients whose imaging data were not used for model development. The breakdown of biomarkers for this group were the following: *IDH1* wildtype/mutant (14/6), *ATRX* wildtype/mutant (16/4), *MGMT* nonmethylated/methylated (10/10), and *EGFR* amplification nondetected/detected (16/4). The overall accuracy of the final (combined conventional-diffusion) models in predicting biomarkers in the test group was 80% for *IDH1* (16/20 were correctly identified), 70% for *ATRX* (14/20 were correctly identified), 70% for *MGMT* (14/20 were correctly identified), and 75% for *EGFR* (15/20 were correctly identified).

## Discussion

Our results showed that constructed multiparametric model from MRI radiomics features can identify IDH1, ATRX, MGMT, and EGFR in preoperative MRI scans of patients with glioma.

We specifically demonstrated that addition of diffusion data to FLAIR and T1c+ can significantly improve predictive performance for *IDH1*, *MGMT*, and *ATRX*, with the highest incremental value achieved for prediction of *MGMT*. MR feature analysis in our study did not contribute in determination of *TP53* or *PTEN* mutational status.

The AUC/diagnostic accuracy of our constructed cross-validated model in prediction of IDH1 status was 0.95/75.8% from conventional MRI features, and was significantly improved to 1.0/79.3% after addition of MR diffusion features. Prior multimodal radiomic studies have predicted *IDH1* status with AUCs ranging from 0.86 to 0.90.^8,9,17,28^ Within LGGs, Eichinger et al.^29^ demonstrated *IDH1* status prediction with AUC of 0.92 using DWI features. *IDH1* wildtype status has been associated with poor survival outcome regardless of WHO grade.^30^ Furthermore, aggressive surgical tumor resection has not been shown to provide survival benefit, specifically in *IDH1* wildtype gliomas.^31^ Therefore, preoperative identification of *IDH1* status can play an important role with prognostic and treatment implications.

Within anaplastic astrocytomas, *ATRX* mutation is a favorable prognostic biomarker associating with longer survival outcome and *ATRX* wildtype has been found to associate with recurrence.^32^ To date, only one prior study has assessed radiomic prediction of *ATRX* status.^14^ They demonstrated predictability with AUC of 0.94 in LGGs using T2-weighted images. Interestingly, a recent study by Ren et al.^15^ showed radiomic prediction of co-occurrence of mutations in *IDH1* and *ATRX* with AUC of 0.93 by combining texture features from FLAIR and DWI images in LGGs. The AUC/diagnostic accuracy of our constructed model in prediction of ATRX status was 0.92/76.9% from conventional MRI features, and was significantly improved to 0.99/80.1% after addition of MR diffusion features. A strong predictive performance is promising as *ATRX* mutation is becoming increasingly recognized as an important prognostic biomarker and is now incorporated in the decision-making algorithm for differentiating oligodendroglial and astrocytic gliomas in the 2016 WHO classification.

For prediction of *MGMT* methylation status, we showed a modest predictive performance for the conventional MRI model with AUC of 0.64 and diagnostic accuracy of only 51.1%. The predictive performance was significantly improved in the combined model after addition of diffusion MRI features with resultant AUC of 0.79 and overall diagnostic accuracy of 67.4%. Prior studies have shown prediction of *MGMT* status with AUC as high as 0.85.^10,11^ Methylation of *MGMT* gene has been associated with longer overall survival and favorable prognostic indicator of response to temozolomide and radiotherapy.^6^ However, subsequent studies have reported conflicting results of the prognostic implication of *MGMT* methylation independent of therapy.^33^*MGMT* promoter methylation has been associated with mitotic counts and Phospho-histone-H3 values as measures of cellular proliferation.^34^ Diffusion MRI provides information about extracellular‐space tortuosity, tissue cellularity, and the integrity of cellular membranes and, therefore, has been used to draw association with *MGMT* status with some success.^35^ It is therefore plausible that addition of diffusion texture features likely exploited the existing differences between *MGMT*-methylated versus nonmethylated groups to explain the highest incremental added value in prediction accuracy obtained by adding diffusion to conventional features in our study.

The AUC/diagnostic accuracy of our constructed model in prediction of EGFR was 0.77/68% from conventional MRI features with no significant incremental improvement after addition of diffusion data. *EGFR* signal amplification has been shown to be a common feature of GBMs^36^ and is associated with aggressive phenotype and poor prognosis.^3^ Li et al.^12^ showed excellent radiomic prediction of *EGFR* amplification with AUC of 0.95 in LGGs. However, there is scarcity of data regarding radiomic prediction of *EGFR* amplification status in GBMs.

Finally, we showed no radiomic prediction of *TP53* or *PTEN* status in either the conventional or combined model. *TP53* is one of the most commonly deregulated genes in cancers and its pathway is deregulated in up to 85% of gliomas.^37^ To date, only Zhang et al.^17^ have assessed radiomic association with *TP53* mutation reporting AUC of 0.95 in LGGs through multimodal combination of features from T1, T2, and FLAIR images. *PTEN* is a tumor suppressor gene significantly altered in 30%–40% of GBM and strongly associated with poor survival.^16^ The loss of *PTEN* function has been mechanistically linked to metastasis and a lack of response to radiotherapy and chemotherapy.^2^ Only one study to date, Li et al.,^16^ has assessed radiomic prediction of *PTEN* status showing excellent AUC of 0.93 by combining texture features from T1 and T2 images.

Establishing an accurate glioma biomarker prediction through radiogenomic approach enables noninvasive prediction of prognosis and contributes to treatment planning. Several studies have assessed individual glioma biomarker status using texture data from either conventional or diffusion imaging, however, only a few studies^9,38^ have combined conventional and diffusion texture data to predict for biomarker status. Qin et al.^39^ combined these features but only in relation to glioma grade without assessing individual glioma biomarkers. Understanding the mutation status of individual glioma markers is critical to guiding treatment.

Our study has several limitations. First, it was a retrospective study with potential for unknown bias. We acknowledge that our patient cohort had a skewed distribution consisting predominantly of GBMs. A prospective study would also allow us to recruit relatively balanced distribution of biomarker status although not all biomarkers statutes can be evenly distributed in the same cohort. Another challenge is the heterogeneous nature of gliomas, especially in GBMs, which vary across individual patients and spatially within each tumor. Thus, biomarker profiles may vary depending on the site of biopsy even within the same tumor, and comprehensive biomarker landscape may not be captured by biopsy alone. As biopsy results were used as gold standard in this study, the innate heterogeneity can introduce inconsistency and potentially inaccuracy which may underestimate the accuracy of our prediction algorithm. Our inability to develop models to predict TP53 or PTEN should therefore be interpreted in the context of this limitation. Techniques assessing tumor purity and multiple gene expression profile^40^ have been used to remedy this limitation, although not available for every patient in our study. Another limitation similar to other radiomic studies is the risk of overfitting considering a large number of variables included. We tried to mitigate this by adopting LASSO regularization followed by logistic regression to select contributing variables while minimizing the potential risk of overfitting and collinearity.^41^ Finally, although preprocessing steps such as signal normalization and resampling were performed to mitigate the effect of image variability related to different MR scanners (*n* = 7) with magnetic fields and vendors, our study was a single center study, external testing of the developed model through multi-institutional collaboration will improve the generalizability and clinical utility of our model.

It should be noted that tumor segmentation was performed by using the entire volume of T2-FLAIR to increase inclusivity. Segmentation on enhancing tumor only as being reported in prior studies,^10,17^ would have resulted in exclusion of 19 out of 111 patients in our study. Furthermore, similar to prior reports,^8,11,39,42^ we included the entire tumor volume on T2 FLAIR images including the cystic and necrotic components to make our approach more generalizable and limit manual segmentation variability. However, texture data may differ when compared with studies excluding these components.^8,10,24^ Further investigation is needed to assess whether inclusion or exclusion of these components may yield significant difference in biomarker prediction.

In conclusion, the described multiparametric MR texture model from combining conventional and diffusion features can predict individual glioma biomarker status in preoperative gliomas. In particular, addition of MR diffusion to conventional MRI features provided significant added diagnostic value in determination of IDH1, MGMT, and ATRX status.

## Supplementary Material

vdab051_suppl_Supplementary_Data

## Notes

**Authorship Statement.** Study design: S.K., N.M.T., J.H., F.K., A.H., D.H., and K.N. Data collection: S.K., N.M.T., Y.S., J.H., F.K., and A.H. Statistical analysis: A.B., N.Z., and K.N. Manuscript writing: S.K., K.N., and N.S.
