# Supplementary material for: Multiparametric MRI texture analysis in prediction of glioma biomarker status: added value of MR diffusion
Source: Neurooncol Adv. 2021 Apr 8;3(1):vdab051. doi: 10.1093/noajnl/vdab051 (PMC8156980; doi:10.1093/noajnl/vdab051)
Supplement: vdab051_suppl_Supplementary_Data [file vdab051_suppl_supplementary_data.docx]

**Supplementary Data:**

**Supplementary Table 1**:

List of contributing texture features from conventional MRI (FLAIR, T1c+) following LASSO regularization and logistic regression analysis

**IDH-1:**

FLAIR – First Order Skewness

FLAIR – GLCM Informal Measurement Correlation 2

FLAIR – GLCM Maximum Probability

FLAIR – GLSZM Small Area High Gray

T1c+ – First Order Skewness

T1c+ – GLCM Difference Variance

T1c+ – GLSZM Size Zone Non-Uniformity Norm

T1c+ – GLSZM Gray Level Variance

T1c+ – GLSZM Small Area High Gray

T1c+ – GLCM Dependence Variance

**MGMT:**

FLAIR – First Order Mean Absolute Deviation

T1c+ – GLCM Cluster Shade

**ATRX:**

FLAIR – GLCM Informal Measurement Correlation 2

FLAIR – GLSZMGL Non-uniformity

FLAIR – GLDM Small Dependence High Gray

T1c+ – GLCM Cluster Shade

T1c+ – GLCM Difference Variance

T1c+ – GLCM Large Dependence High Gray

**EGFR:**

FLAIR – First Order Skewness

FLAIR – GLSZM Small Area Emphasis

T1c+ – GLDM Small Dependence Low Gray

**Supplementary Table 2**:

List of final contributing texture features from conventional MRI (FLAIR, T1c+) and diffusion MRI following LASSO regularization and logistic regression analysis

**IDH-1:**

Conventional

FLAIR – GLCM Informal Measurement Correlation 2

T1c+ – First Order Skewness

T1c+ – GLCM Difference Variance

T1c+ – GLSZM Small Area High Gray

T1c+ – GLCM Dependence Variance

Diffusion

B1000 – First Order Skewness

ADC – First Order Skewness

ADC – GLRLM Run Length Non-Uniformity

ADC – GLSZMGL Non-Uniformity Norm

ADC – GLSZM Small Area High Gray

**MGMT:**

Conventional

FLAIR – First Order Mean Absolute Deviation

T1c+ – GLCM Cluster Shade

Diffusion

B1000 – GLCM Auto-Correlation

B1000 – GLCM Cluster Shade

ADC – GLCM Sum Entropy

**ATRX:**

Conventional

FLAIR – GLCM Informal Measurement Correlation 2

FLAIR – GLCM Dependence Variance

T1c+ – GLCM Difference Variance

T1c+ – GLCM Large Dependence High Gray

Diffusion

B1000 – First Order Skewness

B1000 – GLCM Informal Measurement Correlation 2

B1000 – GLSZM Small Area Emphasis

B1000 – GLSZMN Non-Uniformity Normalized

ADC – First Order Skewness

ADC – GLCM Informal Measure Correlation 1

ADC – GLSZM Gray Level Variance

ADC – GLDMGL Dependence Non-uniformity

**EGFR:**

FLAIR – First Order Skewness

FLAIR – GLSZM Small Area Emphasis

T1c+ – GLDM Small Dependence Low Gray

**Supplementary Figures:**

**Supplementary Figure 1.** Flowchart of study population.


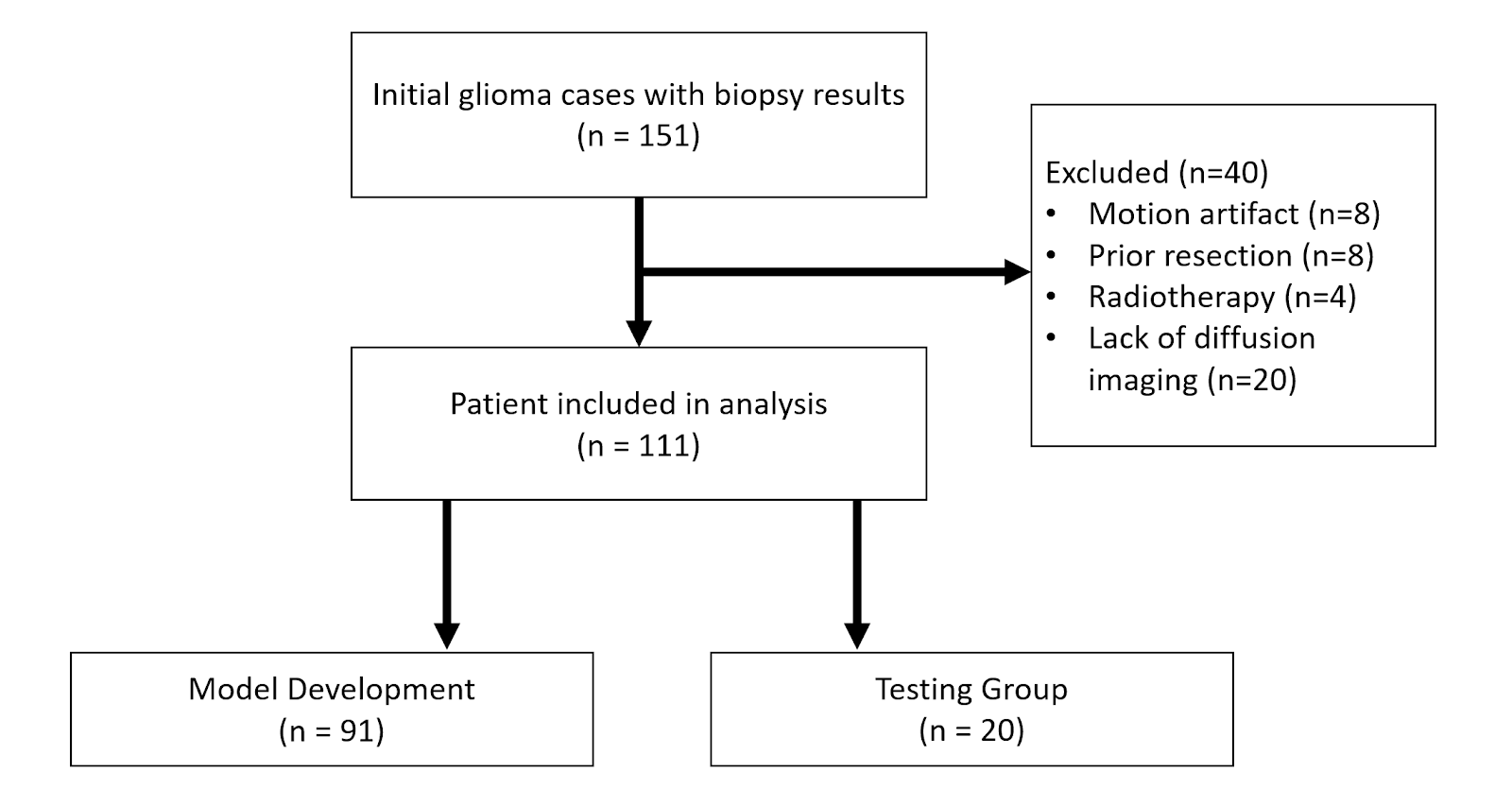


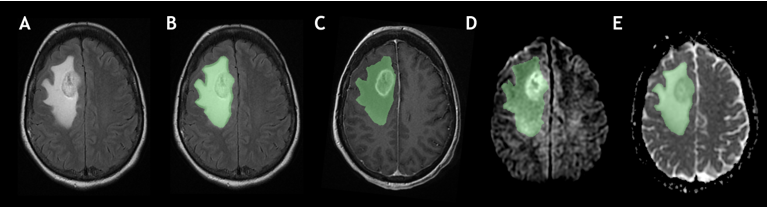


**Supplementary Figure 2.** Example of tumor segmentation. Axial images from a preoperative MRI are shown in a 65-year-old woman with WHO grade IV glioblastoma with biomarker profile of *IDH1* wildtype, *MGMT* methylation, *ATRXATRX* wildtype, *TP53* wildtype, *PTEN* mutation and *EGFR* amplification. Using FLAIR images (A), a volume of interest (VOI) was generated using a voxel-based signal intensity threshold method subsuming the entire region of FLAIR hyperintensity (B). This VOI was then overlaid onto coregistered T1c+ (C), diffusion-b1000 (D) and ADC map (E).
